# Beclin1 circulating levels and accelerated aging markers in COPD

**DOI:** 10.1038/s41419-017-0178-1

**Published:** 2018-02-05

**Authors:** Frédéric Schlemmer, Laurent Boyer, Thibaud Soumagne, Audrey Ridoux, Christos Chouaid, Bernard Maitre, Sophie Lanone, Serge Adnot, Etienne Audureau, Jorge Boczkowski

**Affiliations:** 1 INSERM U955, Institut Mondor de Recherche Biomédicale and Université Paris Est-Créteil (UPEC), Faculté de Médecine, Créteil, France; 2 Unité de Pneumologie, APHP, Hôpital Henri Mondor, DHU-ATVB, Créteil, France; 3 Département de Physiologie-Explorations Fonctionnelles, APHP, Hôpital Henri Mondor, DHU-ATVB, Créteil, France; 4 Département de Pneumologie et Pathologie Professionnelle, Centre Hospitalier Intercommunal, DHU-ATVB, Créteil, France; 5 Département de Santé Publique, Unité de Recherche Clinique (URC-Mondor), APHP, Hôpital Henri Mondor, IMRB EA7376, Clinical Epidemiology and Aging (CEpiA), Créteil, France

To the Editor:

Chronic obstructive pulmonary disease (COPD), so far simply defined by persistent airflow limitation mostly due to prolonged tobacco smoking exposure, is now clearly depicted as a heterogeneous and complex disease, with lung but also systemic manifestations such as sarcopenia, osteoporosis, or cardiovascular diseases^1^. Chronic exposure of lung cells to cigarette smoke can trigger the activation of several cellular processes such as oxidative stress, cellular senescence and autophagy. Induced in response to various stressful situations (e.g. starvation, hypoxia, DNA damage, or infection), autophagy is a major cellular adaptive pathway that, to a certain extent, helps maintain cellular homeostasis through phagolysosomal self-degradation of supernumerary or defective organelles and proteins^2^. Indeed, autophagy theoretically promotes cellular survival but can also favor cell death when this adaptive process is overwhelmed. The implication of autophagy in the pathogenesis of COPD initially looked controversial, with some data showing autophagy activation that favors apoptotic death of bronchial epithelial cells^3^, and some other demonstrating a defective autophagy that may promote cellular senescence^4^. However, recent studies uncovered the link between cigarette smoke-induced oxidative stress, autophagy-flux impairment, accumulation of autophagic vacuoles/aggresome-bodies, chronic inflammatory-apoptotic responses, premature senescence, and emphysema progression in the context of chronic cigarette smoke exposure or in COPD patient’s lung tissue^5–7^. Although there are still areas of uncertainty, like specific steps and mechanisms by which autophagy is impaired, strategies aiming to counteract these phenomena have already emerged as a way to prevent or limit the consequences of chronic cigarette-smoke exposure^8^.

Molecular pathways triggering autophagy are complex and may involve the class III Bcl-2 interacting protein (Beclin1)/phosphoinositol-3-kinase complex, which then participates in the induction and the initial steps of autophagy^2^. A recent study showed that healthy centenarians have increased circulating Beclin1 protein levels in comparison to a population of young healthy subjects or patients with myocardial infarction^9^. Thus, as it has been shown that the induction of autophagy may promote longevity, the authors have suggested a relationship between the increased level of this potential biomarker of autophagy and the exceptional longevity of these patients.

Given that cellular senescence is involved in COPD pathophysiology and that autophagy defect may be a trigger of this process, we hypothesized that circulating Beclin1 levels, taken as a reflect of autophagy process efficiency, are reduced in COPD patients. We therefore tested whether circulating Beclin1 levels are reduced in COPD patients and if so, if this reduction is linked to telomere shortening, a hallmark of senescence. Finally, as deficient autophagy may also be implicated in many pathophysiological processes such as cardiovascular diseases, neuromuscular disorders, or bone loss, we also tested whether Beclin1 level is linked to systemic manifestations of COPD. For that, we took advantage of a cohort of 301 participants recruited at the Henri Mondor Teaching Hospital, Creteil France (COPD, *n* = 100; smokers *n* = 100 and non-smoker patients *n* = 101), already thoroughly phenotyped with a special focus on aging-related markers such as arterial stiffness (aortic pulse-wave velocity, PWV), bone mineral density (BMD), appendicular skeletal muscle mass (ASMM), pinch and grip strengths, insulin resistance, renal function, and telomere length in circulating leucocytes^10^. In 280 (COPD *n* = 92, smokers *n* = 93, non-smokers *n* = 95) of the 301 patients for whom a serum sample was available, we performed a quantitative evaluation of circulating Beclin1 protein level (ELISA Kit for Beclin1; cat. no. E98557Hu, Uscn Life Science Inc., Wuhan, China). Descriptive results are given as numbers and percentages for categorical data, and means (±standard deviation) for continuous variables. Unadjusted comparisons of Beclin1 levels between study groups were conducted using one-way ANOVA for overall significance, and post hoc *t*-tests for pairwise comparisons applying Sidak correction for test multiplicity. Linear regression was used to compare Beclin1 levels across the three groups while adjusting for age. Associations between Beclin1 and biological parameters were assessed by computing Pearson correlation coefficients. All analyses were performed using Stata 14.1 (StataCorp, USA).

## Results

**Fig. 1:**
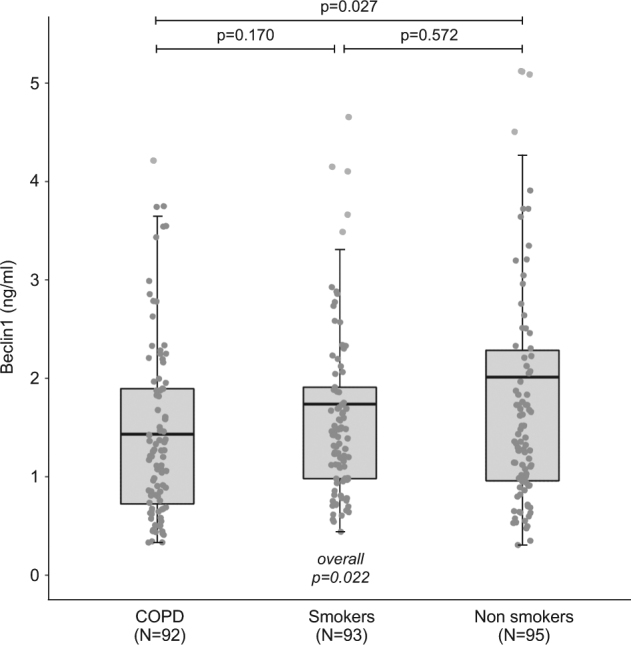
Beclin1 levels according to the study groups. Results are shown as boxplots, with each box representing the interquartile range (1st to 3rd quartile, IQR), the line within the box indicating the mean, and the whiskers extending to 1.5 times the IQR above and below the box; the dots represent individual values for each patient

Clinical characteristics (except pulmonary function tests) did not differ across the three groups, but COPD patients had a higher pack-year value compared to control smokers. A statistically significant negative trend was found in serum Beclin1 protein level of non-smokers, smokers, and COPD patients (*p* = 0.022; Fig. 1). Beclin1 protein level was correlated to age, degree of airway obstruction, telomere length, appendicular skeletal muscle mass index, and grip strength (Table 1). None of the 27 chemokines and growth factors tested was correlated to Beclin1 level. After adjustment for age, the statistically significant negative trend between serum Beclin1 protein level in each group persisted (non-smokers: 2.31 ± 0.23, smokers: 1.66 ± 0.23, and COPD: 1.52 ± 0.24 ng/mL, respectively; *p* = 0.036). In COPD patients, Beclin1 protein level was correlated to pulse-oxygen saturation (*R* = 0.25; *p* = 0.024), telomere length (*R* = 0.26; *p* = 0.014), and TNF-α levels (*R* = 0.35; *p* = 0.001).

**Table 1:** Main subjects characteristics and Pearson correlation coefficients with Beclin1 level

		*N*	Mean (±SD)	Correlation coefficient with Beclin1 level	*p*-value*
Age, years		279	59.02 (±7.76)	**−0.13**	**0.027**
BMI, kg/m^2^		279	26.03 (±4.69)	0.08	0.182
Pack-years		276	27.61 (±27.62)	−0.10	0.088
Pulmonary function parameters					
FEV1, % predicted		279	86.56 (±29.20)	**0.12**	**0.039**
FVC, % predicted		278	93.59 (±21.49)	0.07	0.263
DL_CO_, % predicted		227	76.12 (±19.19)	−0.01	0.906
PaO_2_, mmHg		91	77.47 (±10.32)	0.18	0.086
SpO_2_, %		239	96.59 (±1.35)	0.09	0.178
6-min walking distance, m		239	556.37 (±100.56)	0.05	0.475
Systemic manifestations					
Pulse wave velocity, m/s		271	11.55 (±2.32)	−0.04	0.488
ASMMI, kg/m^2^		270	7.49 (±1.34)	**0.21**	**0.001**
BMD, total lumbar, g/cm^2^		275	1.11 (±0.18)	0.03	0.619
BMD, hips, g/cm^2^		275	0.98 (±0.14)	0.10	0.108
Grip test, kg		244	37.47 (±12.26)	**0.13**	**0.046**
Glomerular flow rate, mL/min		261	93.34 (±48.78)	0.02	0.802
HOMA-IR		265	2.65 (±2.81)	0.05	0.465
Biological parameters					
Telomere length (T/S) ratio		274	0.42 (±0.11)	**0.17**	**0.005**
IL-6, pg/mL		271	18.07 (±15.97)	−0.01	0.905
IL-8, pg/mL		271	46.94 (±9.88)	−0.09	0.155
TNF-α, pg/mL		271	89.52 (±134.53)	0.07	0.228
MCP-1, pg/mL		271	41.66 (±22.44)	−0.02	0.800
		*N*	*N* (%)	Mean (±SD)	*p*-value
Gender	Men	279	185 (66.31%)	1.80 (±1.45)	0.230
	Women		94 (33.69%)	1.58 (±1.40)	
FEV1, % predicted	≥50	279	241 (86.38%)	**1.80 (**±**1.50)**	**0.035**
	<50		38 (13.62%)	**1.27 (**±**0.73)**	

*p-values for Pearson correlation coefficients; bolded results are statistically significant at the *p* < 0.05 level

*BMI* body mass index, *FEV1* forced expiratory volume in 1 s, *% predicted* percentage of the predicted value, *FVC* forced vital capacity, *SpO*_*2*_ oxygen saturation by pulse oximetry, *ASMMI* appendicular skeletal muscle mass index, *BMD* bone mineral density, *HOMA-IR* homeostatic model assessment of insulin resistance, *IL* interleukin, *TNF-α* tumor necrosis factor alpha, *MCP-1* monocyte chemotactic protein-1, *SD* standard deviation

## Discussion

We show a significant decrease in serum Beclin1 protein level, a key regulator of autophagy, in smokers and even more in COPD patients compared to healthy controls. Autophagy induction and apoptosis have been widely described in bronchial and alveolar epithelial cells exposed to cigarette smoke extracts in vitro and in lung tissues of COPD patients^3^. However, evidence on the implication of Beclin1 in the cellular consequences of cigarette smoke exposure are limited to its capacity to permit autophagic-dependant apoptosis induced by acute cigarette smoke exposure^11^. Our results suggest another potential role of Beclin1 in the regulation of autophagy in the specific context of chronic cigarette-smoke exposure. Indeed, accumulation of autophagic vacuoles/agresomes observed in the lungs of patients with severe COPD^3^ already reflects an acquired defect in autophagic process during COPD, attributed to an autophagy-flux impairment that may be accessible to corrective therapies to prevent cellular senescence and emphysema^8^. Thus, Beclin1 circulating levels decline might also participate to autophagy impairment during COPD.

Furthermore, we show a correlation between the level of circulating Beclin1 and the degree of airflow obstruction, which is consistent with a progressive defect in autophagy during COPD, and also with two features of accelerated aging already associated to COPD, cellular senescence^12^ and muscle wasting^10^, respectively evaluated by telomere length, ASMM and grip strength. Of course, the exact relationship between autophagy and cellular senescence remains unclear^13^, especially in the specific context of COPD, and the real implication of autophagy in skeletal muscle dysfunction is still debated^14^. Beclin1 has a pivotal role in the regulation of cell survival, apoptosis, and autophagy, especially via its interaction with anti-apoptotic proteins of the Bcl-2 family (Bcl-2, Bcl-XL, Bcl-w, and Mcl-1), the BH3 domain of Beclin1 important for the binding to the BH3 domain of the pro-apoptotic factors Bak, Bad, and Bim to Bcl-XL, and the activation of the Beclin1-interacting complex that generates phosphatidylinositol-3-phosphate, which promotes autophagosomal membrane nucleation and is indispensable for autophagy^15^. It now seems necessary to further investigate its potential regulatory role in the process of accelerated aging associated to chronic cigarette smoke exposure and COPD, at lung but also at systemic level.

In conclusion, although our results need to be confirmed in other cohorts of COPD patients or with other potential biomarkers of autophagy, they support the hypothesis of a relationship between autophagy deficiency and COPD pathogenesis. They also incite to refine our knowledge on the complex mechanisms linking defective autophagy and cellular senescence in the progressive pathogenesis of COPD and of its systemic manifestations, with a special focus on Beclin1.
